# In Vitro Co-Culture Models of Breast Cancer Metastatic Progression towards Bone

**DOI:** 10.3390/ijms17091405

**Published:** 2016-08-25

**Authors:** Chiara Arrigoni, Simone Bersini, Mara Gilardi, Matteo Moretti

**Affiliations:** 1 Cell and Tissue Engineering Laboratory, IRCCS Istituto Ortopedico Galeazzi, via Galeazzi 4, 20161 Milano, Italy; chiara.arrigoni@grupposandonato.it (C.A.); simone.bersini@grupposandonato.it (S.B.); mara.gilardi@grupposandonato.it (M.G.); 2 Department of Biotechnology and Biosciences, PhD School in Life Sciences, University of Milano-Bicocca, 20126 Milano, Italy; 3 Regenerative Medicine Technologies Lab, Ente Ospedaliero Cantonale, 6900 Lugano, Switzerland; 4 Swiss Institute of Regenerative Medicine, 6900 Lugano, Switzerland; 5 Fondazione Cardiocentro Ticino, 6900 Lugano, Switzerland

**Keywords:** bone metastasis, breast carcinoma, in vitro models, co-culture

## Abstract

Advanced breast cancer frequently metastasizes to bone through a multistep process involving the detachment of cells from the primary tumor, their intravasation into the bloodstream, adhesion to the endothelium and extravasation into the bone, culminating with the establishment of a vicious cycle causing extensive bone lysis. In recent years, the crosstalk between tumor cells and secondary organs microenvironment is gaining much attention, being indicated as a crucial aspect in all metastatic steps. To investigate the complex interrelation between the tumor and the microenvironment, both in vitro and in vivo models have been exploited. In vitro models have some advantages over in vivo, mainly the possibility to thoroughly dissect in controlled conditions and with only human cells the cellular and molecular mechanisms underlying the metastatic progression. In this article we will review the main results deriving from in vitro co-culture models, describing mechanisms activated in the crosstalk between breast cancer and bone cells which drive the different metastatic steps.

## 1. Introduction

Around 70% of patients with advanced breast cancer present skeletal metastases, which cause pain, pathological fractures and an overall decrease of patient quality and the expectancy of life [1]. Despite significant advances in the cure of breast cancer, secondary skeletal lesions remain an unsolved issue, and available specific therapies directed against bone metastases do not significantly increase patient survival as compared to standard chemotherapy [2]. In this scenario, it becomes evident how new effective therapies are needed, counteracting the development of secondary tumors. The spreading of hematogenous metastases is a complex, multistep process, originating with the acquisition of an aggressive, mesenchymal-like phenotype by a subpopulation of cells in the primary tumor, which enters the vasculature, becoming circulating tumor cells (CTCs), and reaches the target organ, transported by the bloodstream [3]. CTCs can then arrest on the endothelium, transmigrate through it (extravasation) and colonize the target organ [4]. Why the bone represents an attractive site for breast cancer metastases is still a matter of debate, and extensive literature exists investigating the mechanisms underlying the preferential metastatization of breast cancer to bone [5,6].

Researchers exploited complimentary methodologies in the effort to elucidate molecular events driving the metastatic spread, and in vivo models represent the most used tool to gain insights into cancer progression [7]. However, even if in vivo models present unquestionable advantages, primarily the recapitulation of the metastatic process in a full, living organism, they also begin to show important limitations, regarding differences in biological mechanisms due to differences between species [8], low control on experimental variables and scarce resolution of applicable analytical methodologies [9]. On the other hand, even if they are a simplified representation of cancer complexity, in vitro models can represent a powerful tool to complement in vivo studies, allowing a thorough dissection of molecular mechanisms, in highly controlled conditions, possibly using only human cells and allowing one to apply single-cell resolution analytical methodologies [10].

**Figure 1:**
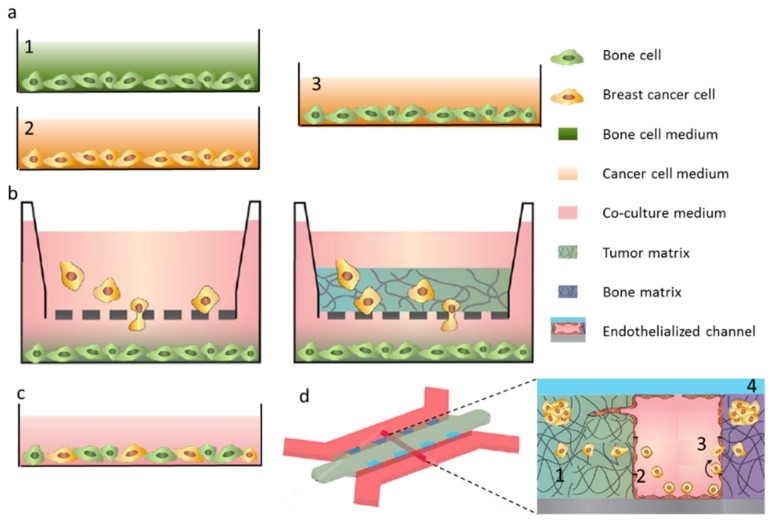
Schematics of different co-culture methods. (**a**) Conditioned medium culture. Culture medium from population (**2**) is used to culture population (**1**), originating an indirect, monodirectional co-culture system (**3**); (**b**) left: Transwell co-culture: population 1 is seeded in the bottom of a culture dish and population 2 is cultured on a porous insert, allowing the exchange of soluble factors, migration of cells through the membrane but without contact between the two populations; right: Transwell can also be used to measure invasion, by coating the porous membrane with a layer of a protein matrix; (**c**) 2D direct co-culture system, in which the two populations are mixed and seeded on the bottom of a culture plate, with a shared medium; (**d**) an example of advanced co-culture systems, allowing one to recapitulate (**1**) the initial migration of cells from the primary tumor, (**2**) intravasation, (**3**) adhesion and extravasation through the endothelium and (**4**) the growth of the metastasis. Adapted from [14].

Historically, the first examples of in vitro cancer models were represented by bi-dimensional cultures of immortalized cancer cell lines [11], used as a simple testing method to screen the ability of candidate drugs to stop cancer cell growth [12]. However, in recent years, the role of the microenvironment in cancer progression received increasing attention, since several studies demonstrated that the reciprocal crosstalk between cancer cells and host cells governs cancer cell behavior, also in the context of metastatic cascade [13]. Thus, as a means to model the interactions between cancer and host cells, co-culture systems have been proposed, ranging from bi-dimensional, indirect co-cultures [14] up to the more recent systems based on complex 3D environments embedding multiple cell types [15]. The simplest co-culture model is represented by the use of conditioned medium: the two cell populations are cultured separately, and the culture medium of one population is collected and used to feed the other cell population (Figure 1a). The main disadvantage of this system is the impossibility to study the bi-directional crosstalk among cancer and bone cells, since only soluble factors released in the medium from one population have effects over the other population.

To overcome this limitation, Transwell systems have been developed, allowing the simultaneous culture of two different cell types sharing the same culture medium but without direct contact (Figure 1b). In Transwell assays, one cell population is seeded on the bottom of a culture plate and the other is seeded over a porous membrane, allowing cell migration in the lower compartment. Reciprocal, paracrine interactions between cells can be studied with this widely used technique, mainly in the context of chemotactic migration. Furthermore, to study cell invasion, the porous membrane can be coated with a layer of a protein gel which is degraded by invading cells that can successively migrate through the membrane (Figure 1b). However, heterotypic interactions caused by direct contact between cancer cells and bone cells are not present, thus researchers exploit also direct co-culture systems (Figure 1c). These traditional 2D models have been extensively used to investigate molecular mechanisms at the basis of cancer metastasis, however they are limited by the simplifications introduced, being the cancer environment characterized by three-dimensionality, presence of multiple cells and of biophysical stimuli [14]. Thus, to more closely replicate the cancer environment, advanced in vitro systems have been recently developed, implementing three-dimensionality, structural organization of host cells and presence of flow (Figure 1d). Yet, these models are still in their initial stages of development and in the majority of cases articles only report descriptive results, for the validation of the model itself, rather than the investigation of biological mechanisms [16,17].

In this context, our review will focus on the main biological findings obtained with the application of co-culture models between breast cancer cells and bone cells, to highlight the contribution of engineered in vitro models to the comprehension of bone metastatic process. We searched in PubMed for “breast cancer bone metasta* AND (co-culture OR coculture)”, limiting the citations to the last ten years. The resulting papers were categorized following the stage of metastatic cascade studied in the paper. We refer the reader to recent excellent reviews for comprehensive descriptions of in vivo models for breast cancer bone metastases [18] and models based on bone slices [19].

## 2. Early Steps of Metastatic Dissemination

**Figure 2:**
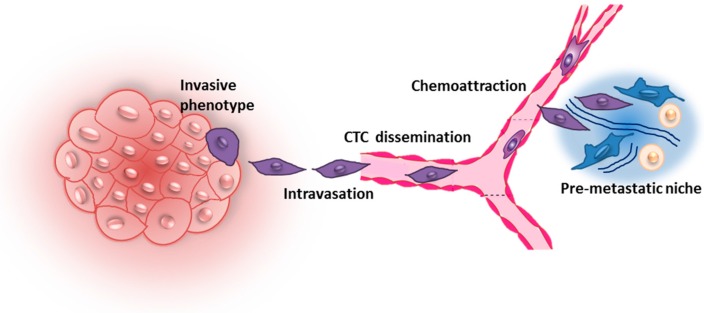
Schematic of the initial phases of metastatic dissemination. A subset of cells of the primary tumor acquires an aggressive phenotype and can detach from the tumor, entering the vasculature. Circulating tumor cells are subjected to chemotactic attraction towards favorable microenvironments called pre-metastatic niches, where they can extravasate and start to form a secondary tumor. Modified with permission from [3] Chaffer, C.L.; et al. A perspective on cancer cell metastasis. *Science* **2011**.

In vitro models involving the co-culture between breast cancer cells (BCCs) and bone cells have been exploited to study the mechanisms driving the metastatic process since the very initial events. As stated in the introduction, the metastatic spreading of a tumor begins with the acquisition of an aggressive phenotype by a subset of cells which allows them to detach from the primary tumor and migrate towards a secondary organ (Figure 2) [3].

Cancer is a very heterogeneous disease, and several subtypes of breast cancers can be defined, with different tendencies to form metastases into bone [20]. The identification of markers that can predict if a tumor is likely to form bone metastases is then fundamental for the classification of patients based on metastatic risk. One of the features that varies between different types of breast cancer is the response of the tumor to estrogen, that is defined by the presence and type of estrogen receptors (ER-α or ER-β) on tumor cells. To investigate a possible link between the hormone receptor status of the primary tumor and its tendency to form metastases in the bone, in vitro co-culture models between BCC lines with different estrogen receptor types and bone cells have been proposed [21,22].

In particular, Sasser and co-workers [21] demonstrated by means of direct co-culture that bone marrow stromal cells (BMSCs) can induce the acquisition of a more aggressive phenotype in ER-α-positive BCCs (MCF-7). Thanks to the in vitro model, which allowed using both human-derived and mouse-derived BMSCs, it was possible to demonstrate that IL-6 produced by human, but not by mouse-derived BMSCs was able to induce phosphorylation of STAT-3 and subsequently to increase the growth rate of MCF-7, discovering a specific mechanism underlying the higher bone metastatic potential demonstrated in advanced ER-α-positive tumors [23]. On the other hand, it has been demonstrated that ER-α, but not ER-β, -positive cells in co-culture with bone cells increased their expression of osteopontin, indicating the acquisition of an osteo-mimetic phenotype, which can also be related to the probability of metastasizing to the bone [22]. In the same study, genomic analysis of the two different BCCs subtypes after co-culture was carried out to evidence estrogen-responsive genes, which can dictate a more aggressive phenotype. Among others, the genes that were more expressed in ER-α- and ER-β-positive BCCs were Muc-1 and MacMARCKS, known to be related to cell-cell and cell-matrix adhesion [24] and to cell adhesion and spreading [25], respectively. It can be concluded that the expression of a specific hormone receptor subtype influences the expression of genes involved in the early detachment of BCCs from primary tumor and in the acquisition of a phenotype more prone to metastasize to bone, thus representing potential prognostic markers to identify high-risk patients.

Apart from these few examples of direct co-culture between breast cancer and bone cells, the majority of works investigating the early steps of metastatic spreading focus on the chemotactic attraction exerted by bone cells over BCCs. Preferential chemotactic migration towards bone of BCCs is mainly orchestrated by soluble factors released by the bone cells [26]; thus, an indirect co-culture model is more suitable to dissect such paracrine effects. In this context, the system most used is represented by Transwell, together with Boyden chambers and conditioned medium assays.

Transwell assays can be used to measure both BCC migration and invasion. To quantify the latter phenomenon, the Transwell porous membrane is coated with a layer of matrix that can be degraded by the proteolytic action of BCCs [14]. This approach has been used in the work of Pohorelic and colleagues [27], investigating the role of Src kinase in BCCs migration and invasion in the bone. Several BCCs lines were compared, both in co-culture with bone-derived cells and in control conditions, showing that the more aggressive MDA-MB-231 line and co-cultured BCCs compared to respective BCCs in control conditions showed a higher Src kinase-specific activity. Furthermore, the inhibition of Src by a specific inhibitor and by siRNA decreased BCC migration towards bone cells. Interestingly, BCC invasion was not modified by Src inhibition and was not different between BCCs cultured with bone cells or with control fibroblasts, suggesting that BCC invasion is less influenced by the surrounding microenvironment as compared to migration, which is instead greatly influenced by chemoattractants produced by bone cells [26].

One of the major mechanisms underlying the chemotactic attraction of breast cancer cells towards bone has been identified in the CXCR-4/CXCL-12 axis [28], also critical for other bone-metastasizing tumors, such as prostate cancer [29]. CXCL-12 (also called stromal cell-derived factor 1 (SDF-1)) is a well-known chemokine secreted by stromal cells playing a key role in hematopoiesis, driving the homing of hematopoietic stem cells (HSCs) into the bone marrow niche [30]. To clarify upstream pathways involved in the chemotactic migration driven by this chemokine, Guo and co-workers [31] established a Transwell indirect co-culture system between two lines of BCCs (the more invasive MDA-MB-231 and the less aggressive MCF-7) and a bone-like cell line (MG63). They showed that BMP-4-transduced BCC lines migrated more and showed increased expression of CXCR-4. Furthermore, they found an increased expression of CXCL-12 on co-cultured MG63. When an inhibitory factor for BMP-4 was added to the co-cultures, migration of breast cancer cells was decreased and their CXCR-4 expression downregulated, demonstrating that BMP-4 acted as an activator of the CXCR-4/CXCL-12 pathway. Another work [32] confirmed the central role of the same pathway in the chemotactic migration of BCCs towards bone-like cells, demonstrating how the protein Kisspeptin-10, known to inhibit metastasis of different cancer types, such as melanoma and breast cancer [33], negatively regulated the expression of CXCR-4 and consequently decreased MCF-7 migration towards MG63 bone cells. The CXCR-4/CXCL-12 axis seems to be regulated also through estrogen receptors. Indeed, it has been demonstrated [34] that the addition of an ER-β receptor agonist to three different estrogen-responsive BCC lines, indirectly co-cultured with MG63, caused a decrease in their migration and in CXCR-4 expression. Thus, the results of the study suggested that the selective activation of ER-β receptors led to the reduced metastatic potential of BCCs.

Another cytokine that has been involved in the chemotactic migration of BCCs towards bone is CCL-2 (also called monocyte chemotactic protein (MCP-1)), known to regulate the attraction of various immune cells to inflammation sites and to activate monocytes [35]. CCL-2 is secreted by several cell types, and it has been shown that BMSCs differentiating towards osteoblasts produced increasing amounts of the protein as osteodifferentiation advances [36]. Furthermore, when BCCs were put in indirect co-culture with osteo-differentiated BMSCs, their migration rate increased, and the effect was partly abolished by the addition of a CCL-2 monoclonal antibody, suggesting an important role of this cytokine in BCC migration. Further evidences confirming the potential role of MCP-1 as a chemoattractant come from the study of Bussard and co-workers [37]. Although the study presented some limitations due to the use of mouse bone-derived cells co-cultured with human cancer cells, it confirmed how soluble factors (among which MCP-1 and IL6) produced by bone cells are chemotactic for BCCs and that the cross-talk between BCCs and bone cells alters the production of molecules by bone cells.

This concept of dynamic and reciprocal interactions between BCCs and bone cells has been recently resumed in the theory of pre-metastatic niches [38]. In this view, tumor cells guide the formation of a pre-metastatic niche by secreting a plethora of cytokines and growth factors that in turn promote mobilization and recruitment of BMSCs towards future metastatic sites, to create a favorable environment for tumor cell engrafting. To study such a complex phenomenon, recent studies have been published, based on indirect co-culture models, aiming at identifying key molecules, cells and ECM proteins, which constitute the target niche in the bone marrow [39,40].

ECM proteins have been indicated as important factors determining tumor cell behavior [41], and among them, tenascin W has attracted special interest due to its abundant presence in the niche of osteoblastic progenitors [42]. To study the potential involvement of tenascin W in the establishment of a pre-metastatic niche, BCCs were co-cultured in a Transwell system with BMSCs, and BCC migration, expression of tenascin W and its transcriptional regulation were analyzed [39]. It has been found that BCCs in co-culture (both direct and indirect) with BMSCs induced the expression of tenascin W in BMSCs, and this effect was mediated by soluble substances secreted by BCCs. On the contrary, BMSCs did not elicit the production of tenascin W in BCCs, highlighting how the role of this molecule is restricted to the metastatic microenvironment. To discover which soluble factors released by BCCs caused the production of tenascin W, a transcriptional regulation analysis was performed, demonstrating that TGF-β1 secreted by BCCs induced SMAD-4-dependent transcription of the tenascin W gene in BMSCs. To confirm that TGF-β1 was regulating the protein production, TGF-β1 receptors on BMSCs were blocked, decreasing tenascin W production. As a further indication of the importance of this ECM protein specifically for the metastatic bone niche, the researchers demonstrate how the conditioned medium from a line of BCCs preferentially metastasizing to bone (MDA-MB231-1833) increased the production of tenascin W in BMSCs as compared to the conditioned medium from a parental BCC line (MDA-MB231). Besides increasing the production of tenascin W, TGF-β1 has been demonstrated to foster the generation of pre-metastatic niches in the bone marrow also through different mechanisms. Wobus and co-workers [40] recently reported that TGF-β1 produced by BCCs can alter the gene expression profile and, consequently, the secretion of key molecules by BMSCs, and in particular, exposition of BMSCs to medium conditioned by BCCs decreased the production of CXCL-12 by BMSCs. The addition of a monoclonal antibody against TGF-β1 completely restored CXCL-12 production by BMSCs. Since, as already stated, CXCL-12 is involved in the homing of HSCs in the bone marrow, a decreased production of the chemokine can alter the balance between circulating and resident HSCs. Indeed, in the peripheral blood of patients with breast cancer, a higher number of HSCs were found, suggesting how tumor cells can hijack physiological mechanisms that assure stromal support to stem cells, to create a favorable microenvironment, which will facilitate their homing in the target organ.

Despite the relevant findings obtained with co-culture models, it has to be evidenced that further improvements are possible, to facilitate the translation towards effective clinical treatments. In particular, the study of early events originating BCC bone metastases could be more relevant if obtained in the context of a 3D tumor model. Furthermore, the presence of flow in chemotaxis assays could better replicate the in vivo BCC behavior. Finally, in the majority of cited co-culture systems only two cell types are co-cultured, while the presence of several other cell types (endothelial cells, osteoclasts) can add a substantial contribution to the observed mechanism.

## 3. Breast Cancer Cell Extravasation to Bone—In Vitro Modeling of Key Mechanisms

The extravasation step of the metastatic cascade represents the last key event before the invasion of secondary tissues and the establishment of micrometastases. Cancer cells flowing into the bloodstream can become physically trapped within small capillaries or they can roll and adhere on the endothelium. In the latter scenario, the initial attachment is mediated by endothelial selectins and cancer cell counter-receptors, while subsequent stable interactions with the endothelium involve the active role of integrins and other receptors, including CD-44 and mucins [4]. Then, cancer cells transmigrate through the endothelial wall in a process that is often mediated by organ-specific cytokines, which attract cancer cells [43].

Several in vitro modeling approaches characterized by a different level of complexity can be adopted to recapitulate the process of cancer cell extravasation. These ones span from traditional Transwell assays to recent microfluidic systems, which can mimic transendothelial migration through either endothelial monolayers or physiological-like microvessels [15]. Surprisingly, only a few in vitro models have specifically analyzed the mechanisms driving BCCs extravasation to bone tissues.

Corcoran and co-authors employed Transwells with a double layer of BMSCs and bone marrow endothelial cells (ECs) to study the signaling promoting the extravasation of BCCs with different levels of metastatic potential. In particular, a lower number of transmigration events was detected without BMSCs, and this reduction was higher for BCCs with low metastatic potential (T47D and MCF-7 vs. MDA-231), suggesting that BMSCs can differentially modulate extravasation according to the invasive ability of cancer cells. Specific BCCs express surface-bound SDF-1α and the chemokine receptor CXCR-4, whose knock-down (KD) was demonstrated to influence both adhesion and transmigration. In addition, the authors found that Tac-1 KD, which is associated with reduced expression of both SDF-1α and CXCR-4, was able to impair adhesion and transmigration and that re-expression of CXCR-4 (but not SDF-1α) in Tac-1 KD cells partly (MDA-231) or completely (TD47) restored BCC ability to migrate through the endothelial monolayer [44]. Despite this model representing one of the first attempts to analyze the mechanisms driving organ-specific metastases of BCCs to bone, it did not allow reproducing the complete process of cancer cell flow, attachment, transmigration and colonization of the bone. Furthermore, Transwell assays do not allow real-time monitoring of adhesion/transmigration events nor controlling the biochemical/biophysical stimulation of the local microenvironment.

**Figure 3:**
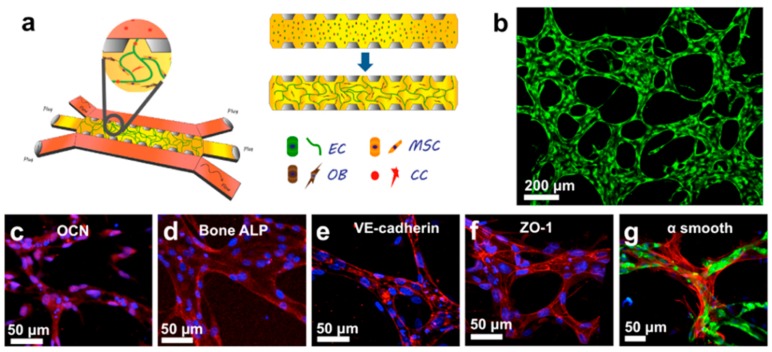

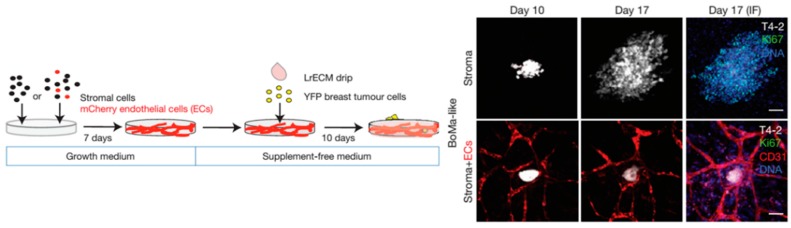
**Upper** panel: in vitro modeling of cancer cell extravasation and early invasion. (**a**) Microfluidic model of breast cancer cell (BCC) extravasation towards a bone-mimicking microenvironment containing mesenchymal stem cells (BMSCs) and osteo-differentiated BMSCs (OBs); (**b**) perfusable microvascular networks (green) allowing cancer cell flow, adhesion and trapping within capillary-like structures; (**c**–**g**) Representative figures showing expression of bone and vascular specific markers (red), namely osteocalcin (OCN, **c**), bone alkaline phosphatase (ALP, **d**), vascular endothelial (VE)-cadherin (**e**), zonula occludens (ZO)-1 (**f**) and α smooth muscle actin (**g**). Endothelial cells (ECs): green; nuclei: blue. Reproduced by permission from [47] Jeon J.S.; et al. Human 3D vascularized organotypic microfluidic assays to study breast cancer cell extravasation. *Proc. Natl. Acad. Sci. USA* **2015**; **Lower** panel: engineered model for the study of the effect of microvasculature on BCC dormancy. **Left**: BMSCs were seeded alone (stroma) or in co-culture with ECs (microvasculature niche). BCCs were seeded onto stroma or microvasculature niche and laminin rich ECM (LrECM) was deposited to create a 3D environment for cancer cell study. YFP: yellow fluorescent protein; **Right**: T4-2 BCCs seeded on bone marrow (BoMa)-like stroma (scale bar: 100 μm) or BoMa-like stroma + ECs (scale bar: 50 μm) showing how the presence of microvasculature significantly reduced the presence of Ki-67 positive cells and induced a dormant state. T4-2 cells: white; Ki67: green; CD31: red; nuclei: blue. Reprinted by permission from [48] Ghajar, C.M.; et al. The perivascular niche regulates breast tumour dormancy. *Nat. Cell Biol.* **2013**.

Microfluidic models allow one to overcome these limitations, thus representing promising tools to analyze the molecular mechanisms driving BCC extravasation. Recently, our group has developed two microfluidic models to study BCC transmigration/colonization of a bone-mimicking microenvironment generated with hydrogel-laden osteo-differentiated BMSCs. In the first simplified model, microfluidic channels containing BMSCs embedded in a collagen matrix were endothelialized, and MDA-231 cells were flowed through these biomimetic microvessels, showing a preferential transmigration towards the bone-like matrix compared to empty collagen matrix. More in detail, it was demonstrated that the CXCL-5-CXCR-2 signaling axis was involved in the extravasation process, since gradients of CXCL-5 generated through the microfluidic device were able to attract BCCs to control acellular matrices, while antibodies blocking CXCR-2 significantly reduced BCC transmigration towards bone-mimicking microenvironments. Interestingly, the addition of CXCL-5 not only affected BCCs transmigration, but also their migration distance once extravasated [45]. Despite this system representing the first microfluidic model mimicking the organ-specificity of BCCs metastases to bone, it did not fully recapitulate the transmigration of cancer cells through capillary-like vessels nor the effects of physiological flows. Microvascular networks were then developed within fibrin matrices containing osteo-differentiated BMSCs and mural-like BMSCs, which wrapped around microvessels (Figure 3, upper panel). BCCs (BOKL, bone seeking clone of MDA-231) were then infused into the perfusable microvessels and extravasated towards the bone-mimicking microenvironment, while a control muscle-mimicking matrix was not able to attract cancer cells. Several molecules can be involved in the anti-metastatic features characterizing the skeletal muscle. In particular, the muscle-secreted adenosine was demonstrated to reduce bone metastases in vivo through interaction with the A3 adenosine receptor, which is expressed by BCCs [46], despite its role in extravasation not being previously clarified. Surprisingly, the addition of adenosine was able to reduce BCC extravasation to the bone-mimicking microenvironment, despite increasing vascular permeability. Conversely, blocking the A3 adenosine receptor (PSB-10 antagonist) on BCCs injected in the muscle-mimicking microenvironment increased BCC transmigration. These data clearly demonstrated that muscle-secreted adenosine was able to impair BCCs extravasation, but also showed how endothelial permeability was not the key factor driving extravasation [47].

## 4. Bone Tissue Colonization

### 4.1. From Early Invasion to Cancer Cell Dormancy

Once extravasated, cancer cells must adapt to the local microenvironment and tune their features through bi-directional interactions with organ-specific cells. The bone tissue is particularly rich in cytokines, hormones and growth factors, whose release can be conditioned by the interaction with BCCs, hence promoting the progression of metastases [49].

Rajski and co-authors developed a simple co-culture model to study the cross-talk between BCCs and osteoblasts with the aim to find a correlation between the gene signature of these heterotypic co-cultures and the expression profile of human tumors in vivo. In particular, the authors found that IL-6 mRNA was significantly higher in MDA-231-osteoblast co-cultures compared to the sum of the two mono-cultures. IL-6 is a cytokine involved in a wide set of processes, including cell proliferation, angiogenesis and inflammation. When compared to human samples, the presence of IL-6 was positively correlated with bone metastases and a lower metastasis-free survival at 10 years [50].

Another example of the interaction between bone cells and BCCs was reported by D’Ambrosio and Fatatis, who investigated how osteoblasts can modulate the calcium signaling in BCCs. Cells are generally characterized by low cytosolic calcium levels, while deregulation of this condition may drive cytotoxicity and cell death. The authors exposed low (MDA-468) and high (MDA-231) metastatic cancer cells to ATP, which induces an increase in intracellular calcium followed by a plateau. The behavior of highly metastatic BCCs was divided into groups characterized by high or low plateau levels of intracellular calcium. The authors found that co-cultures of BCCs and osteoblasts promoted a shift towards low plateau responses, but the removal of osteoblasts reverted cancer cell behavior. Thus, the presence of osteoblasts tunes the calcium signaling in BCCs, protecting them from the conditions of cellular stress. Interestingly, similar results to those obtained with osteoblast co-cultures were shown by treatment with histone deacetylase, demonstrating that osteoblasts reversibly mediate calcium signaling in metastatic cancer cells through this enzyme [51].

It is important to highlight that cancer cell invasion of the local microenvironment does not represent an effective and straightforward process. Indeed, most extravasated cancer cells do not survive in the newly-colonized microenvironment, and the rate of cancer cells able to induce the formation of stable macrometastases is extremely low [52]. Disseminated tumor cells often survive in the secondary tissue in a condition of cell-cycle arrest named cellular dormancy [53].

A striking example of the cellular dormancy model was developed by Ghajar and co-authors, who hypothesized that ECs and the basement membrane could be responsible for the generation of a dormant niche. ECs and BMSCs were co-cultured to generate a primitive microvasculature, and BCCs were subsequently seeded on their top, finding that the presence of ECs significantly reduced the growth of BCCs, including T4-2, MCF-7 and MDA-231, as shown in Figure 3, middle panel. Significantly, cancer cells associated with a stable microvasculature were characterized by a dormant state, and perivascular thrombospondin (TSP-1) was responsible for this condition. Conversely, TSP-1 was downregulated at neovascular tips, which were generally associated with proliferating BCCs. Furthermore, networks with a higher number of tip cells and branch points showed higher expression of active transforming growth factor (TGF)-β1, while stalk cells preferentially expressed latent TGF-β1 [48].

Similar results were found by Marlow and colleagues, who developed 3D co-cultures models of inhibitory niches (osteoblasts, mesenchymal and endothelial cells) or supportive niches (bone marrow stromal cells and BCCs; Figure 3, lower panel). Noteworthy, BCCs were able to re-start proliferating once extracted from the inhibitory niche. These results show that dormancy can be directly modulated by the local microenvironment, while specific cancer cell signaling pathways can be inhibited to block cellular dormancy (e.g., p38 MAPK or TGF-β) [53]. Zhou et al. also reported that co-cultures of BCCs and BMSCs induced cancer cell dormancy with more cells entering the G0 or G1 phase compared to the S phase. This dormant state seems to be promoted by BMSC-secreted SDF-1α, which alters Tac-1 expression and reduces neurokinin receptor 1 (NKR1) [54]. Noteworthy, Tac-1 KD was previously shown to reduce BCC extravasation, suggesting that the same gene can be involved in different steps of the metastatic cascade [44]. Ono and colleagues extended the established paradigm that BCC-BMSC co-cultures induce cancer cell dormancy, demonstrating that co-cultures significantly reduce the expression of stem-like markers, cell invasion (matrigel assay) and sensitivity to chemotherapeutics. A key finding of this study was the identification of the same effects when BCCs were cultured with BMSC-secreted exosomes. In particular, the authors demonstrated that miR-23b was transferred to BCCs leading to the suppression of MARCK-5, which is involved in cell motility and cell cycling [55]. The transfer of miRNA from bone marrow stromal cells to colonizing BCCs is emerging as a key aspect of cellular dormancy. Indeed, miRNA 127, 197, 222 and 223 were transferred from the bone marrow stroma to BCCs through gap junctions, leading to reduced CXCL-12 and impaired cancer cell proliferation [56]. Noteworthy, Lim and co-authors confirmed that miRNA transferred through exosomes contribute to the dormant state, despite their effect being less pronounced compared to the gap junction communication system [56].

### 4.2. Metastasis Growth and Formation of Osteolytic Lesions

Up to now, it is not clear why, even after several years, BCCs exit from the dormant state and start to grow, originating an overt metastasis. Recently, bone remodeling cytokines produced in inflammatory processes consequent to traumatic events have been indicated as potential activators of dormant metastatic cells [57]. With a 3D co-culture model between the dormant BCC line MDA-MB-231BRMS1 and fetal osteoblast MC3T3-E1, the authors demonstrated that dormant BCCs did not proliferate in co-culture until TNF-β and IL-α were added, suggesting that modifications in the bone microenvironment due to inflammatory cytokines may regulate the insurgence of latent bone metastases.

**Figure 4:**
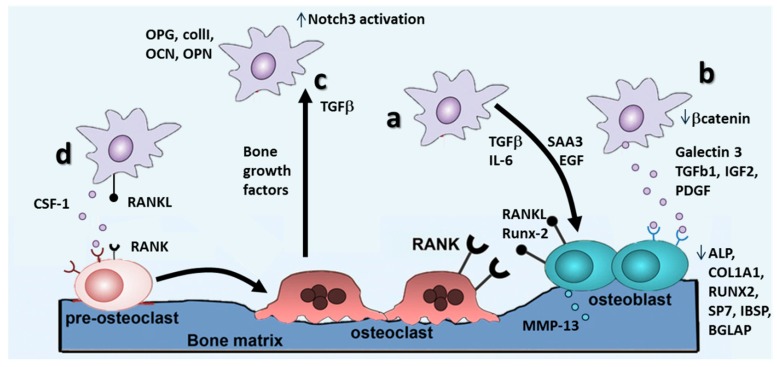
Mechanisms involved in the establishment of osteolytic bone metastases. (**a**) Effects of BCCs on bone resorption mediated by osteoblasts: molecules secreted by BCCs (TGF-β, IL-6, EGF, etc.) have been shown to activate osteoblasts to produce MMP-13 that degrades bone matrix and RANKL and RUNX-2, which stimulate osteoclasts to resorb bone; (**b**) direct effects of BCCs on osteoblasts differentiation: molecules secreted by BCCs (Galectin-3 and TGF-β1, IGF-2, PDGF) and BCC-expressed proteins (β-catenin) have been shown to regulate osteoblastic differentiation (affecting the expression of specific osteoblastic markers such as ALP, RUNX-2, etc.) through different pathways; (**c**) effects of molecules released by bone cells on BCCs: crosstalk between bone and cancer cells has been shown to promote an osteomimetic phenotype in BCCs (increasing the expression of typical bone markers such as osteocalcin OCN, osteopontin OPN, etc.) and TGF-β released by bone cells has been shown to activate Notch3 signaling and to promote BCCs growth; (**d**) direct effects of BCCs on osteoclastic differentiation: molecules secreted (CSF-1) or expressed (RANKL) by BCCs have been demonstrated to promote differentiation and activation of osteoclastic precursors towards mature osteoclasts. ↑: increased; ↓: decreased. Adapted by permission from [61] David, L.W.; Theresa, A.G. Cancer-associated muscle weakness: What’s bone got to do with it. *BoneKEy Rep*. **2015**.

Once rescued from the dormant state, BCCs are known to promote osteolytic bone metastases through the establishment of “vicious cycles” (amplifying feedback loops), which induce continuous bone resorption and release of pro-tumorigenic factors [58,59] (Figure 4). Beyond the well-studied RANKL-RANK-OPG pathway [59] which will be discussed in a separate section, other signaling programs are involved in the formation of an osteolytic milieu. Morrison and colleagues characterized through co-cultures (direct contact, Transwell, conditioned medium) of BCCs (MDA-1833) and osteoblasts which osteoclast-independent features are critical for bone metastases [60]. The authors found a significant increase in matrix metalloprotease (MMP)-13 mRNA production by osteoblasts when co-cultured with BCCs. The presence of MMP-13 was shown to increase the level of CCL-2, platelet-derived growth factor (PDGF)-C and serum amyloid A3 apolipoprotein SAA3, which can promote monocyte recruitment and osteoclast differentiation. Hence, BCCs produce factors, such as SAA3, that induce osteoblast secretion of MMP-13, which in turn activates the inducers of MMP-13, stimulating additional MMP-13 production and the generation of a vicious cycle (Figure 4a).

Besides the creation of a proteolytic milieu, BCCs interfere with bone homeostasis by altering the balance of resident bone cell differentiation, as widely reported in the literature for osteoclasts, but less characterized for osteoblasts (Figure 4b). In this context, Chen and co-workers focused on β-catenin, which plays a key role in skeletogenesis and post-natal bone regeneration, and investigated its potential involvement in the regulation of osteoblast and osteoclast differentiation [62]. Using a direct co-culture between murine BCCs and murine osteoblasts or osteoclasts, the authors observed that β-catenin was upregulated in bone metastatic BCCs (TM40D-MB) as compared to parental BCCs, and inactivation of β-catenin in the bone metastatic line inhibited osteoblast differentiation while increasing osteoclast differentiation. Furthermore, inhibition of β-catenin increased osteolytic bone resorption in vivo, even if BCCs overexpressing β-catenin induced a slight increment of osteoblast differentiation only in vitro, however confirming an involvement of β-catenin in regulating osteoblastic differentiation within the metastatic bone environment. Another emerging factor involved in the BCCs alteration of osteoblasts differentiation has been identified in galectin-3, a tumor-secreted sugar-binding protein, which can activate Notch signaling, involved in embryonic development, differentiation and proliferation [63]. The authors co-cultured breast cancer BT-549 with human fetal osteoblasts, demonstrating an inhibition in osteoblast differentiation as compared to controls. More in detail, they found a downregulation of the expression of osteoblast differentiation markers, such as ALPL, COL1A1, RUNX2, SP7, IBSP and BGLAP, due to galectin-3 secreted by BCCs, which accelerated Notch1 cleavage and activation in a sugar-dependent manner.

Other studies evidenced the importance of Notch signaling in the establishment of BCCs bone metastases, elucidating the role of osteoblast-derived TGF-β1 in Notch3 signaling activation in cancer cells (Figure 4c) [64]. The authors performed direct and Transwell co-culture of bone marrow osteoblasts with the human BCCs line MDA-MET (a bone-seeking clone derived from MDA-MB231), demonstrating that both direct contact and secreted factors from osteoblasts induced a two-fold increase in tumor cells of the expression of Notch3, as well as Notch3 ligand Jagged1. Furthermore, through the soft agar assay, they demonstrated that Notch3 silencing affects osteoblast-induced anchorage-independent growth in cancer cells. Finally, they identified osteoblast-secreted TGF-β1 as the regulator of the overexpression of Notch3 in BCCs. TGF-β has been involved also in the regulation of bone cell differentiation, since it has been reported that TGF-β produced by MDA-MB-231 BCCs inhibited MC3T3-E1 osteoblasts differentiation, morphology, actin stress fiber pattern and reduced focal adhesion plaques. Using neutralizing antibodies against PDGF and insulin-like growth factor II (IGF-II) in conditioned medium from MDA-MB 231, focal adhesion plaques and actin stress fiber formation were restored in co-cultured MC3T3-E1 osteoblasts. Furthermore, they evidenced that these cytokines activate signaling pathways, such as PI3 kinase and Rac, highlighting potential molecules that can be targeted to restore bone homeostasis altered by breast cancer metastases. Recently, TGF-β has been also indicated as a mediator of the pro-metastatic activity of integrin-β-like1 (ITGBL1) in BCCs [65]. The authors characterized the role of ITGBL1 in bone metastatic process, by knocking down and overexpressing this molecule in BCCs lines and monitoring cell migratory potential with a Transwell assay. Results of the work demonstrated that expression of ITGBL1 regulated the migration of BCCs towards bone cells and knockdown of ITGBL1 downregulated bone-related genes in BCCs, indicating a reversal of the osteomimetic phenotype and a consequent decrease of bone-metastatic potential. Furthermore, the overexpression of ITGBL1 in BCCs increased the production of osteoclast-stimulating factors, as confirmed by the increased maturation of murine osteoclast precursors cultured in the presence of medium conditioned by BCCs. When a blocker of the TGF-β SMAD signaling pathway was used, all of the effects of ITGBL1 overexpression were suppressed, suggesting that the TGF-β signaling pathway mediates the role of ITGBL1 in breast cancer bone metastasis.

#### The RANKL/RANK/OPG Pathway

Besides suppressing osteoblasts differentiation, BCCs have been shown to enhance osteoclast differentiation, switching bone homeostasis towards resorption [58] (Figure 4a,d). Osteoblasts derive from mesenchymal stem cells and are recognized as the main sources of factors regulating osteoclasts formation, including receptor activator of NF-κB ligand (RANKL), macrophage colony-stimulating factor (M-CSF), osteoprotegerin (OPG) [66] and monocyte chemoattractant protein 1 (MCP-1) [35]. M-CSF regulates osteoclast precursors in terms of proliferation, survival and differentiation [67], while MCP-1 was shown to stimulate osteoclast fusion and activity [68]. RANKL is the key mediator for osteoclast fusion, differentiation and activation [69], while OPG is the soluble decoy receptor able to block the interaction of RANKL with its receptor RANK expressed on osteoclasts [70]. All of these players strictly regulate bone resorption/deposition balance, making them attractive as therapeutic targets.

To analyze the incidence of soluble mediators secreted by BCCs on osteoclast from human origin in terms of maturation (Figure 4d), an indirect co-culture model was set up [71]. The authors reported that MDA-MB-231 secreted CSF-1, and BCC-conditioned medium increased osteoclastic differentiation. To investigate if this phenomenon were CSF-1 dependent, they analyzed the capacity of 5H4 (an anti-CSF-1 antibody) to impair BCCs ability to promote osteoclast differentiation and activation, evaluated by the TRAP assay. Furthermore, the effects on osteoclasts differentiation of zoledronate, (a third-generation bisphosphonate) and Denosumab (a human anti-RANKL antibody) were investigated. The treatment with both 5H4 and Denosumab reduced osteoclast differentiation and survival, while the exposition to zoledronate induced osteoclast apoptosis. Furthermore, BCCs induced resistance to zoledronate and increased sensitivity to 5H4 in cancer-induced-osteoclasts as compared to osteoclasts differentiated with factors. Direct co-culture models have been widely used, in particular to study the direct effects of BCCs on osteoclasts [72], with the aim to determine if MCF-7 BCCs could induce monocytes differentiation towards osteoclasts. The authors identified RANKL as the effector of BCCs promoted osteoclast differentiation, and in particular, the RANKL trans-membrane isoform, expressed by BCCs, produced an effect on monocytes differentiation, while the soluble form played a minor role, only in early differentiation steps. As a further confirmation that also BCCs express RANKL, another study reported that the direct co-culture with bone cells induced RANKL expression in BCCs [73]. In particular, the authors studied BCCs (BOKL, a bone-seeking clone derived from MDA-MB231) gene expression after direct and indirect co-culture with human BMSCs differentiated toward the osteoblastic lineage. The co-cultures were digested and FACS sorted to obtain separated populations, and PCR analyses were performed on the sorted populations, evidencing a significant increment in the RANKL/OPG ratio only in BCCs after direct co-culture, suggesting a key role of heterotypic cell interactions. This fundamental role of heterotypic junctions between BCCs and bone cells in the establishment of bone metastases has been elucidated also in a recent article [74], showing how BCCs expressing E-cadherin form adherens junctions with osteogenic cells, mainly expressing N-cadherin in a 3D co-culture model. The authors demonstrated that heterotypic adherens junctions between cancer cells and osteoblasts or BMSCs (but not with osteoclasts) were necessary to activate the mTOR pathway in BCCs and, consequently, to elicit a proliferative response of cancer cells in the bone microenvironment, which was completely abolished if the formation of junctions was inhibited.

Besides the activation of osteoclast differentiation mediated by RANKL, BCCs also stimulate osteoblasts to secrete osteoclast-stimulating factors. Zhao and co-workers [75] investigated how BCCs promoted the production of osteoblasts-derived factors stimulating osteoclastogenesis, and moreover, they studied the effects of calcitonin gene-related peptide (CGRP) on co-cultures between MDA-MB 231 BCCs and the MG63 human osteoblast-like cell line. The authors demonstrated that interactions between MDA-MB-231 and MG63 did not affect the survival of osteoblasts, but BCCs caused osteolytic lesions by inducing the upregulation in MG63 of RUNX-2, an essential factor for osteoblast differentiation, which however also mediates osteoclast activation. Furthermore, they showed that CGRP has an opposite effect than that of BCCs, increasing osteoblasts formation and inhibiting osteolysis through the regulation of the RANKL and OPG ratio. RANKL expression in osteoblasts can be also induced by interleukin-6 (IL-6) secreted by BCCs. Indeed, IL-6 expression in serum has been linked with poor prognosis in cancer patients. To better investigate the role of IL-6 in the crosstalk between BCCs and osteoblasts, Zheng and co-workers [76] analyzed if RANKL released by KUSA-O or primary osteoblasts directly induced IL-6 expression in MDA-MB231, using a direct co-culture system. Firstly, they discovered that RANKL from osteoblasts upregulated the secretion of IL-6 by BCCs. Secreted IL-6 in turn induced RANK expression by BCCs, which sensitized the tumor cells to RANKL released from the bone microenvironment and, thus, boosted a positive loop. The confirmation that this auto-amplifying cross-talk was involved in bone metastases was also validated in vivo, highlighting how RANKL and IL-6 are important regulators of direct paracrine and autocrine signaling in bone resorption, leading to increased proliferation of BCCs within the bone metastatic environment. An alternative mechanism by which BCCs stimulate osteoblasts to produce osteoclast-stimulating factors has been identified in epidermal growth factor (EGF) signaling [77]. EGF and related receptors are implicated in the most important signaling pathways in tissue development and cancer biology and have been also linked to bone homeostasis. Using a co-culture system composed of murine osteoclast precursors and MC3T3-E1 murine osteoblasts in which BCCs (MDA-MB231) can be also added, the authors evidenced that EGF-like ligands stimulate osteoclasts differentiation. Since osteoclasts do not express functional EGF receptors, the enhanced osteoclastogenesis could be explained by an indirect effect, mediated by osteoblasts, further confirmed by culturing pre-osteoclasts in medium conditioned by EGF-treated osteoblasts. Furthermore, EGF-like ligands on osteoblasts decreased OPG expression and increased the secretion of monocyte chemoattractant protein 1 (MCP1), factors known to stimulate osteoclastogenesis. To elucidate a possible role of EGF signaling in bone metastasis, the secretion of EGF-like ligands was measured in BCCs, finding a high expression of EGF-like ligands, at least at mRNA level. Taken together, these results evidence that BCCs cross-talk with osteoblasts increases osteoclastogenesis through the EGF signaling pathway, amplifying the release of bone-matrix-trapped factors, which finally fosters tumor growth triggering and feeding the vicious cycle.

While it is well known that an increased RANKL/OPG ratio shifts bone homeostasis towards osteoclastogenesis, less is known about the role of OPG in BCCs. In this context, a peculiar result has been reported by Kapoor and colleagues [78]. They indeed showed that the MDA-231, MDA-435, MDA-MET and MDA-231/K BCCs lines expressed OPG, and its level was directly correlated with metastatic potential and specific bone homing. Furthermore, they highlighted the expression of other bone-related markers in BCCs including type I collagen, osteocalcin, osteopontin and RUNX-2, suggesting that cancer cells can acquire an osteo-mimetic phenotype. However, the higher expression of OPG in bone-metastatic cells was not correlated to a higher expression of other bone markers, suggesting that OPG expression was not merely a consequence of enhanced osteomimicry, but that it had a key role in the metastatic process.

## 5. Conclusions

In conclusion, the application of in vitro co-culture models allowed to discover the fundamental molecular mechanisms driving specific steps of the metastatic progression of breast cancer to bone (Table 1), thanks to the possibility to accurately control the applied environmental conditions and, thus, to dissect the effects of distinct variables, an approach not applicable within in vivo models. Furthermore, the exploitation of high-resolution imaging and quantitative techniques ensures a deep insight into the molecular and cellular mechanisms. However, traditional co-culture models are severely limited by the over-simplifications of the system, sometimes leading to scarce translation into a real clinical benefit. Aiming to overcome this critical issue, advanced in vitro devices are needed, which could better recapitulate key hallmarks of the metastatic cascade. A fundamental characteristic is the three-dimensionality of the in vivo environment, which has been shown to be a key regulator of cell behavior [79] and, thus, would likely play a role also in the metastatic spreading of BCCs to bone. Furthermore, to study CTC arrest on endothelium and extravasation, it is essential to recreate a vascular system similar to physiological capillaries, such as recently described in millimeter-scale engineered bone models [80,81]. A perfusable microvascular network could also allow the inclusion of blood cellular components, which have been indicated as key players for CTC survival in the bloodstream and adhesion to endothelium [82,83]. Finally, striking evidence is mounting against the use of cancer cell lines, which have been started to be considered too dissimilar from heterogeneous real tumors and, thus, fostering the research of alternative cellular sources [84]. In this context, the generation of in vitro tumor models based on patient-derived cells could provide an invaluable contribution, allowing one to investigate the effects of tumor inter-individual heterogeneity on the bone metastatic progression, achieving a significant progress in the field of personalized medicine.

## Figures and Tables

**Table 1:** Summary of the different molecules involved in breast cancer bone metastasis, discovered with the application of different in vitro models.

Step of Metastatic Dissemination	Mechanism Studied	Type of In Vitro Model Used	Molecules Involved	References
Early steps of bone metastasis	Acquisition of bone-aggressive phenotype	2D direct co-culture	IL-6	[21]
			ER-α, ER-β	[22]
	Chemotactic migration	Transwell	Src-kinase	[26]
			CXCR-4/CXCL-12	[31,32,34]
			CCL-2	[36,37]
	Pre-metastatic niche	Transwell, conditioned medium	Tenascin W	[39]
			TGF-β1, CXCL-12	[40]
Extravasation	Transedothelial migration	Transwell	SDF-1α, CXCR-4, Tac-1	[44]
		Microfluidic	CXCR-2/CXCL-5, adenosine	[45,47]
Bone colonization	Early invasion	2D direct co-culture	IL-6	[50]
			Ca^2+^	[51]
	Dormancy	3D direct co-culture	TSP-1, TGF-β, p38	[50,53]
		2D direct co-culture	Tac-1, NRK-1	[54]
			miRNAs from BMCs	[55,56]
	Cancer cell growth in the bone	2D and 3D direct co-culture	ILα, TNF-β	[57]
			E-cadh, N-cadh	[73]
	Interaction with osteoblasts	2D direct co-culture	MMP-13	[60]
			β-catenin	[61]
		Transwell, 2D direct co-culture	Galectin-3, Notch, TGF-β	[62,63]
	Osteoclast maturation	Conditioned medium	Integrin β1, TGF-β	[64]
			CSF-1	[70]
		2D and 3D direct co-culture	RANKL/RANK, CGRP, IL-6	[71,72,74,75]
			EGF, MCP-1	[76]
			OPG	[77]
